# Trends in the contributions of atopic family history to pediatric food sensitization and allergy

**DOI:** 10.3389/fped.2022.967930

**Published:** 2022-12-07

**Authors:** Heping Fang, Zhuoying Ma, Lin Chen, Ruoling Xian, Juan Wang, Jing Chen, Haiqi Li, Yan Hu

**Affiliations:** Department of Child Health Care, Children’s Hospital of Chongqing Medical University, National Clinical Research Center for Child Health and Disorders, Ministry of Education Key Laboratory of Child Development and Disorders, Chongqing Key Laboratory of Child Health and Nutrition, Chongqing, China

**Keywords:** children, family history of atopic diseases (FHA), food allergy (FA), food sensitization (FS), trends

## Abstract

**Objective:**

Family history of atopic diseases (FHA) contributes to food allergy (FA). But little is known whether FHA primarily increases IgE–mediated, non–IgE–mediated FA, or both. And the trends in the contributions of FHA to food sensitization (FS) and FA remain unclear. We aim to clarify the associations among FHA, FS and FA and to understand the trends in the contributions of FHA to FS and FA.

**Methods:**

We used chi–square test and mediating effect model to analyze the associations among FHA, FS and FA through comparisons between two cross–sectional investigations on FA in children under 2 years old in 2009 and 2019.

**Results:**

In 2009 and 2019, the positive FHA proportion tended to be increasing without significance (28.9% to 31.6%, *P *= 0.39). Subgroup analysis showed the FS rate in FA group decreased significantly (37/39 to 44/62, *P *= 0.003). In 2009, the FS rate and FA prevalence were higher in FHA (+) group than in FHA (–) group (26% vs. 14.7%, *P *= 0.008 and 15% vs. 7.7%, *P *= 0.03), and FS had a complete mediating effect on the association between FHA and FA (*Z *= 2.54, *P *= 0.011), but the results lost significance in 2019.

**Conclusions:**

The association between FHA and FA was completely mediated by FS, which means FHA mainly increases IgE–mediated FA. And the contributions of FHA to FS and FA tended to be stabilized or even diminished, which means FHA alone could no longer be enough to screen high–risk children.

## Introduction

Food allergy (FA) means abnormal immune responses and symptoms caused by food antigens, and is more complicated than other atopic diseases as it includes IgE–mediated, non–IgE–mediated and mixed–mediated types. In recent years, the reported incidence of FA has increased (1), and FA has become an important global public health issue and has brought heavy burdens to families and societies (2, 3).

Like other atopic diseases, genetic factors contribute to FA (4). Many genes are involved in FA, such as the nonsense mutation of the filaggrin gene increases the risk of FA (5). Meanwhile, clinical studies have confirmed that family history of atopic diseases (FHA) is also involved in FA. For example, peanut allergy had a strong genetic susceptibility and the estimated heritability (the proportion of variation that is due to hereditary factors) (6) was approximately 81.6% (7). Maternal asthma was significantly associated with cow's milk allergy in children with atopic dermatitis (AD) (OR* *=* *10.9, 95% CI 1.6–73) (8). And the risk of FA increased to 2.6–fold (95% CI 1.2–5.6) in children with FA siblings (9). Therefore, inquiring about the family history is essential (10), and FHA is often used as the indicator of genetic factors to screen children at high risk of FA in clinical practice.

However, little is known about whether FHA primarily increases IgE–mediated, non–IgE–mediated FA, or both. In other words, whether FHA is associated with the development of FA directly or indirectly. It is known that FHA has an impact on IgE production and infants with FHA have increased Th2 cytokines (IL–4, IL–13, etc.) (11, 12), which are the key cytokines in the initiation and chronicity of Th2 immune responses (13, 14). As a consequence, FHA can promote the differentiation of immune system towards Th2 type in children, which may lead to atopic diseases. Moreover, parental atopic respiratory disease increased the risk significantly more for IgE–associated AD (15), and FHA children with food sensitization (FS) might be a susceptible subgroup to FA (16). Thus, we speculate that FS could mediate the association between FHA and FA, that means FHA may mainly increase the risk of FS, and then FA.

More importantly, the FS rate among infants with FHA has remained stable in Australia since the 1990s (17). But the incidence of both IgE–mediated and non–IgE–mediated FA is increasing, which is beyond the range that could be explained by genetic variation (18, 19). This discrepancy could be due to increased FA in the non–FHA population, increased conversion of FS to FA, or increased number of high–risk infants (17). In fact, limited evidence suggests that FHA is even not enough to predict FS or FA in children (20–23), two or more allergic family members or FHA combined with environmental factors may be more effective in screening children at high risk for FA (24, 25), Therefore, the contributions of genetic and environmental factors to the development of FS and FA could have changed over time, suggesting that it is time for allergists to re–evaluate the role of FHA in FS and FA. In this article, we aim to clarify the associations among FHA, FS and FA, and to understand the trends in the contributions of FHA to FS and FA.

## Participants and methods

### Study design

This article was complementary to our previously published study (26). In a word, we conducted three cross–sectional investigations on FA in the Department of Child Health Care, Children's Hospital of Chongqing Medical University (CHCMU) in 1999, 2009, and 2019. As previously described, we had already discussed the FS rate and FA prevalence in the past 20 years (the most common food allergens were cow's milk and egg). Here, we grouped the participants based on FHA into FHA (+) group and FHA (–) group, and analyzed the differences of FS rate and FA prevalence between the two groups to better understand the possible changes in the contributions of FHA to FS and FA. Notably, the data from 2009 to 2019 were included, and the data from 1999 were excluded due to unclear definition of FHA. The article followed the STROBE Statement.

### Participants

All healthy children under 2 years old attending routine physical examinations at the Department of Child Health Care were recruited in the two investigations without selection. All children recruited came from the main nine districts of Chongqing, because they might be recalled for oral challenge test (OFC). Informed consent was obtained upon initial contact. According to the National Health Commission of the People's Republic of China, children should attend at least 6 routine physical examinations within the first 2 years after birth (27), with a community management rate of more than 80% (28). In addition, the Department of Child Health Care, CHCMU is the largest department that carries children's routine physical examinations in Chongqing, with an average annual outpatient volume of 110,000 in the past three years. Therefore, although this study was conducted through a hospital based convenient sample, it still had good community representation (29).

The sample sizes were calculated using a single proportion sample size estimating algorithm (30). The reference value for FA prevalence in 2009 was 3.5% from our 1999 survey, with an allowable error of 2.0%. The reference value in 2019 was 7.7% from our 2009 survey, with an allowable error of 2.5% (26). The confidence level was set as 95%, and the drop–out rate was predicted to be 10%. The calculations provided 362 and 485 as the minimum sample sizes for the two investigations, respectively.

### Investigation and diagnosis procedure

The same survey design was adopted in 2009 and 2019. The diagnosis of FA was performed by questionnaire, skin prick test (SPT), food avoidance test and OFC in sequence. The entire procedure was carried out by trained pediatricians as previously described (26). In a word, SPT was performed in all eligible children after obtaining medical histories. Then, 2-weeks food elimination tests were carried out for those with positive histories or SPT (the mean wheal diameter was at least 3 mm greater than the negative control at 15 min). OFC was performed in the hospital by a trained pediatrician to diagnose FA in children who benefited from food elimination tests. The diagnosis procedure was in accordance with the EAACI guidelines (31). In this article, FHA was defined as any first–degree relative diagnosed with AD, FA, allergic asthma, or atopic rhinitis. And FS and FA were determined by positive SPT and OFC, respectively.

### Data processing and statistical analysis

**Figure 1:**
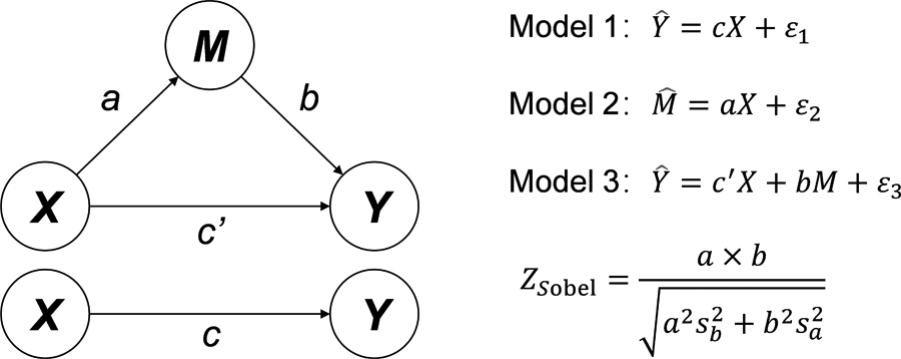
Standard tri–variate mediating effect model and Logistic regression models. X: Independent variable. Y: Dependent variable. M: Mediator.

All data were analyzed by SPSS 26.0 (IBM Corp). The chi–square test was used to analyze the demographic characteristics (age, sex, and positive FHA proportion), FS rate and FA prevalence between the two investigations. The associations among FHA, FS and FA were analyzed by binary Logistic regression according to the mediating effect model of the categorical variables (32, 33). The standard tri–variate mediation model (Figure 1) was adopted to build three binary Logistic regression models: (1) regression of the independent variable (*X*) on the dependent variable (*Y*) (Model 1), (2) regression of *X* on the mediator (*M*) (Model 2), and (3) regression of *X* and *M* on *Y* (Model 3). We applied the method proposed by Sobel (34) to calculate the *Z* value of the mediation model, and the *P* value was obtained from the *Z* table. Statistical significance was defined using a two–sided significance level of *α *=* *0.05, and a *P* value* *<* *0.05 or an absolute *Z* value >1.96 was considered statistically significant.

Participants who failed to follow the entire diagnostic procedure were regarded as drop–outs (all drop–outs in these two investigations did not perform OFC). The demographic characteristics were compared between participants following the diagnostic procedure and drop–outs. The FA prevalence has been reported referring to the intention–to–treat principle (35) as previously described (26). To further discuss the associations among FHA, FS and FA, multiple imputations were performed to create five complete datasets in this study. Each dataset was analyzed separately, and the results were the average of the five datasets. Additionally, the results before and after imputation were compared.

## Results

### Demographic characteristics

In 2009 and 2019, 401 and 513 participants were included with 19 (4.7%) and 10 (1.9%) participants dropping out for personal reasons, respectively. The proportion of participants under 1 year of age in the drop–outs was lower in 2009 (11/19 vs. 306/382, *P *= 0.04), while sex and positive FHA proportion in 2009 and the demographic characteristics in 2019 were not significantly different between these two groups. In addition, the results before and after multiple imputation were not significantly different, and the conclusions were consistent (results not shown).

**Figure 2:**
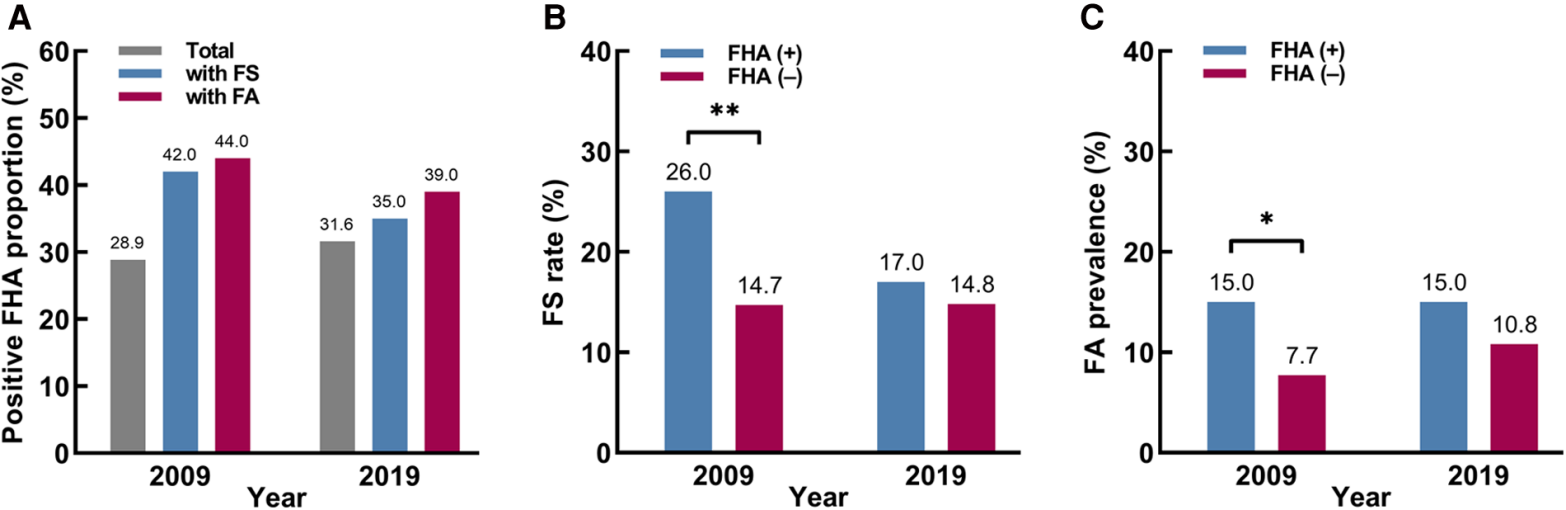
The proportions with a positive family history of atopic diseases (FHA), food sensitization (FS) rates and food allergy (FA) prevalence among different participants. (**A**) The positive FHA proportions among different participants. (**B**) The FS rates based on FHA. (**C**) The FA prevalence based on FHA. * *P *< 0.05. ** *P *< 0.01.

Table 1 shows the demographic characteristics of the two investigations. In 2009 and 2019, 54.4% (218/401) and 52.2% (268/513) of the participants were male, and 79.1% (317/401) and 73.9% (379/513) of the participants were under 1 year of age, respectively. The age and sex in the two investigations were not significantly different (*P *= 0.52 and 0.07). From 2009 to 2019, the positive FHA proportion was 28.9% (116/401) in 2009, and 31.6% (162/513) in 2019, which did not change significantly in different groups, respectively (*P *> 0.05) (Figure 2A).

**Table 1:** Demographic characteristics, and the positive FHA^†^ proportions in different participants in 2009 and 2019.

Characteristics	2009 (*N* = 401)	2019 (*N* = 513)	*χ* ^2^	*P*
Age, *n* (%)				
0 to 1 years	317 (79.1)	379 (73.9)	3.32	0.07
1 to 2 years	84 (20.9)	134 (26.1)		
Sex, *n* (%)				
Male	218 (54.4)	268 (52.2)	0.41	0.52
Female	183 (45.6)	245 (47.8)		
Positive FHA proportion, *n*/*N* (%)				
In total	116/401 (28.9)	162/513 (31.6)	0.75	0.39
In FS^‡^ group	30/72 (42)	28/80 (35)	0.71	0.40
In FA^§^ group	17/39 (44)	24/62 (39)	0.24	0.63

^†^ Family history of atopic diseases.

^‡^ Food sensitization.

^§^ Food allergy.

### FS rate and FA prevalence

Table 2 shows the FS rates and FA prevalence based on FHA in 2009 and 2019. The FS rate tended to be decreasing in FHA (+) group (26% to 17%, *P *= 0.08) but stabilized in FHA (–) group (14.7% to 14.8%, *P *= 0.98). And the FA prevalence tended to be increasing in FHA (–) group (7.7% to 10.8%, *P *= 0.18) but stabilized in FHA (+) group (15% to 15%, *P *= 0.97). Then, the stratified analysis by year showed that in 2009, the FS rate and FA prevalence were higher in FHA (+) group than in FHA (–) group (FS rate: 26% vs. 14.7%, *P *= 0.008 and FA prevalence: 15% vs. 7.7%, *P *= 0.03). However, in 2019, the same trends still existed but lost significance (Figures 2B,C).

**Table 2:** The FS^†^ rate and FA^‡^ prevalence in different participants in 2009 and 2019.

Characteristics	2009 (*N* = 401)	2019 (*N* = 513)	χ^2^	*P*
FS rate, *n*/*N* (%)				
In FHA (+) group	30/116 (26)	28/162 (17)	3.01	0.08
In FHA (–) group	42/285 (14.7)	52/351 (14.8)	<0.01	0.98
FA prevalence, *n*/*N* (%)				
In FHA (+) group	17/116 (15)	24/162 (15)	<0.01	0.97
In FHA (–) group	22/285 (7.7)	38/351 (10.8)	1.78	0.18

^†^ Food sensitization.

^‡^ Food allergy.

In addition, the FS rate in participants with FA decreased significantly (37/39 to 44/62, *P *= 0.003). Notably, all the allergens which caused symptoms were tested by SPT with negative results.

### The associations among FHA, FS and FA

FHA, FS and FA were included in the standard tri–variate mediation model as *X*, *M* and *Y*, respectively. Before analysis, age and sex were examined between different FHA groups to determine whether to be included in the model for adjustment (Supplementary Table S1). Model 1 and Model 2 showed that in 2009, FHA had significant associations with FA and FS (*P *= 0.046, *P *= 0.006), respectively. However, the association between FHA and FA in Model 1 lost significance after adding FS into the model (Model 3). The *Z* value was 2.54 with a *P* value of 0.011, indicating that FS had a complete mediating effect on the association between FHA and FA in 2009; in other words, the association between FHA and FA was mediated by FS. Interestingly, in 2019, Model 1 and Model 2 showed that the associations between FHA and FA or FS lost significance (Table 3).

**Table 3:** Mediating effect model of FHA^†^, FS^‡^ and FA^§^.

Model	Regression	B	*β*	Wald	OR (95% CI)	*P*
2009 (adjusted for sex)						
Model 1	FHA on FA	0.76	0.38	4.00	2.14 (1.01–4.54)	0.046
Model 2	FHA on FS	0.75	0.27	7.44	2.11 (1.23–3.61)	0.006
Model 3	FHA on FA	0.16	0.49	0.11	1.17 (0.45–3.06)	0.75
	FS on FA	4.72	0.76	38.94	112.58 (25.53–496.38)	<0.001
2019						
Model 1	FHA on FA	0.36	0.28	1.72	1.44 (0.83–2.49)	0.20
Model 2	FHA on FS	0.18	0.26	0.51	1.20 (0.73–1.99)	0.47
Model 3	FHA on FA	0.37	0.34	1.21	1.45 (0.74–2.85)	0.29
	FS on FA	3.37	0.33	103.21	29.02 (15.17–55.53)	<0.001

^†^ Family history of atopic diseases.

^‡^ Food sensitization.

^§^ Food allergy.

## Discussion

### Principle findings

Epidemiological studies on atopic diseases show that the overall incidence has been increasing in the past 20 to 30 years (36, 37), which will increase the proportion of FHA–positive children. According to our data, infants with positive FHA accounted for 28.9% in 2009 and 31.6% in 2019. It seemed that the positive FHA proportion in China was lower than in Western countries (58.6% to 69.0%) (24, 38). But the proportion could be lower than the actual level due to the lack of allergists and insufficient knowledge about atopic diseases in general pediatricians and parents in China (39), which may lead to missed diagnoses of atopic diseases. Interestingly, although without significance, the increasing trends in positive FHA proportion appeared to be inconsistent with the decreasing trends in FS rate. It suggested that in some ways, the contributions of FHA to FS and FA may have changed over time.

More importantly, we found that the association between FHA and FA was completely mediated by FS, and the contributions of FHA to FS and FA tended to be stabilized or even diminished. The role of FHA in FS and FA is already confirmed (7, 8), but the associations among FHA, FS and FA remain unclear. Thus, we conducted a mediating effect analysis to further examine the associations from a population perspective. As we speculated, the results showed that FS had a complete mediating effect on the association between FHA and FA in 2009, which means FHA mainly increases IgE–mediated FA. It is consistent with a few other studies (15, 16), which can be explained by the fact that FHA can promote the differentiation of immune system towards Th2 type (12), culminating in the production of IgE (40).

Interestingly, the associations between FHA and FS or FA lost significance in 2019, suggesting that the contributions of FHA to FS and FA could have changed over time. Moreover, our 2009 data showed that the FS rate and FA prevalence in FHA (+) group were significantly higher than in FHA (–) group, but the differences also lost significance in 2019 (Figures 2B,C). This seemed to be due to the decreasing trends in FS rate in FHA (+) group and increasing trends in FA prevalence in FHA (–) group. These results suggest that there is a need to re–evaluate the role of FHA in the development of FS and FA. In recent years, some studies have already focused on the effect of FHA on FS and FA. In 2013, Goldberg et al (20). reported that parental atopy (positive SPT) was not a risk factor for persistent IgE–mediated cow's milk allergy in children. Then, in 2015, Nissen et al. (21) found that isolated FHA was not a strong predictor for sensitization and allergic symptoms from childhood until early adulthood. Later, Gupta et al. (22) showed that in FA families, only a small proportion of siblings were both sensitized and symptomatic to food (13.6%), while asymptomatic sensitization was more common (53.0%). Most recently, Keet et al. (23) indicated that family history of peanut allergy did not confer substantial risk in the absence of moderate–severe eczema (only 1% of infants with FHA and no eczema had peanut allergy). All these studies suggest that FHA alone is not sufficient to predict FS or FA in children, which is basically consistent with our study. Moreover, our results further suggested it may not be that FHA could not predict FA, but that the contributions changed to no longer sufficient for prediction. Therefore, it is reasonable to assume that the contributions of FHA to FS and FA could have stabilized or even diminished.

Furthermore, our results showed that FA was significantly associated with FS both in 2009 and 2019 (Table 3), suggesting that the change in the contributions of FHA was mainly between FHA and FS rather than between FS and FA. The change could be absolute (the contributions of genetic factors decreased) or relative (the contributions of environmental factors increased). That means some robust environmental factors could have influenced the production of IgE, and concealed the contribution of genetic factors. For example, urbanization has large–scale effects on the human microbiota (41), and some bacteria (staphylococcus aureus, etc.) do release virulence factors as superantigens to promote IgE production (42), which will undoubtedly cause sensitization and allergy (43–45). Our results showed that the FS rate in FA group decreased significantly, and given the continuous increase in FA incidence and the effects of environmental factors (18, 46), we prefer that the change is more likely to be a relative change caused by environmental factors. Therefore, the contributions of environmental factors will be interesting to elucidate.

### Limitations

This study had some limitations. First, this study did not conduct stratified analysis on FHA by different relatives or atopic diseases, which could bring some bias in using FHA as an indicator of genetic factors. Second, although selection bias may be not significant as the children recruited were attending for a routine physical examination, it could not be ruled out because CHCMU is the largest children's medical center in western China, which may attract children from families with better economic conditions in the community. Third, the sample size of our study was relatively small, and the possibility of a type II error could not be definitely excluded. Finally, the environment of the children may have changed in the past ten years, resulting in potential bias.

## Conclusion

In conclusion, our data demonstrated that the association between FHA and FA was completely mediated by FS, and the contributions of FHA to FS and FA tended to be stabilized or even diminished, which is more likely to be a relative change caused by the increasing contributions of environmental factors. This is a meaningful finding that FHA alone could no longer be enough to screen infants at high risk for FS or FA. Allergists may need to re–evaluate the role of FHA in FS and FA, and combine FHA with environmental factors to screen high–risk children. Notably, the present findings were preliminary, which should be reproduced in other studies using different population before the conclusion is established.

## Data Availability

The original contributions presented in the study are included in the article/**Supplementary Material**, further inquiries can be directed to the corresponding author/s.
