# Supplementary material for: Trends in the contributions of atopic family history to pediatric food sensitization and allergy
Source: Front Pediatr. 2022 Dec 7;10:967930. doi: 10.3389/fped.2022.967930 (PMC9768553; doi:10.3389/fped.2022.967930)
Supplement: Supplementary file 1 [file Table1.docx]

| **Supplementary Table 1. Age and sex in different FHA^†^ groups in 2009 and 2019.** | | | | |
| --- | --- | --- | --- | --- |
| **Characteristics** | **FHA (+)** | **FHA (–)** | ***χ^2^*** | ***P*** |
| **2009 (N=401)** | | | | |
| **Age, n (%)** | | | | |
| 0 to 1 years | 90 (77.6) | 227 (79.6) | 0.21 | 0.65 |
| 1 to 2 years | 26 (22.4) | 58 (20.4) |  |  |
| **Sex, n (%)** | | | | |
| Male | 74 (63.8) | 144 (50.5) | 5.85 | 0.016 |
| Female | 42 (36.2) | 141 (49.5) |  |  |
| **2019 (N=513)** | | | | |
| **Age, n (%)** | | | | |
| 0 to 1 years | 124 (76.5) | 255 (72.6) | 0.87 | 0.35 |
| 1 to 2 years | 38 (23.5) | 96 (27.4) |  |  |
| **Sex, n (%)** | | | | |
| Male | 83 (51.2) | 185 (52.7) | 0.10 | 0.76 |
| Female | 79 (48.8) | 166 (47.3) |  |  |
| ^†^ Family history of atopic diseases. | | | | |
